# EEG criticality as a prognostic tool for functional outcomes in sedated pediatric intensive care patients

**DOI:** 10.3389/fncom.2026.1831476

**Published:** 2026-07-08

**Authors:** Derek Newman, Mark Grinberg, Kevin Jones, Kristine Woodward, Michael J. Esser, Stefanie Blain-Moraes

**Affiliations:** 1 Integrated Program in Neuroscience, McGill University, Montreal, QC, Canada; 2 Montreal General Hospital, McGill University Health Centre, Montreal, QC, Canada; 3 Department of Pediatrics, Faculty of Health Sciences, McMaster University, Hamilton, ON, Canada; 4 Department of Pediatrics, Cumming School of Medicine, University of Calgary, Calgary, AB, Canada; 5 School of Physical and Occupational Therapy, McGill University, Montreal, QC, Canada; 6 Department of Occupational Sciences and Occupational Therapy, University of British Columbia, Vancouver, BC, Canada; 7 School of Biomedical Engineering, University of British Columbia, Vancouver, BC, Canada

**Keywords:** anesthesia, criticality, electroencephalography, neuroprognosis, pathological brain states, pediatric

## Abstract

**Objective:**

Predicting meaningful recovery in sedated patients remains a major challenge in the pediatric intensive care unit (PICU) due to the lack of reliable, behavior-independent prognostic markers for children. Criticality of electroencephalography (EEG) signals reflects the brain’s dynamic balance between order and chaos and capacity for information processing. The objective of this study was to assess the association between criticality-related EEG features and the functional outcomes of sedated PICU patients.

**Methods:**

A multi-center, retrospective cohort observational study was conducted with 32 patients admitted to two PICUs in urban Canada between 2014 and 2024, 5–18 years of age, 14 females. Patients were admitted with mixed etiology: acute seizure (28%), acute brain injures (22%), and systemic illness (50%). All patients received a clinically indicated EEG while exposed to an inhibitory anesthetic (midazolam, propofol, dexmedetomidine with a GABAergic sedative). Nine EEG features were calculated from three categories of criticality-related measures (entropy, fractal and complexity); five spectral EEG features and three patient demographic features were extracted from the databases. Patient outcomes were assessed with the Glasgow Outcome Scale-Extended three months post-injury. All features were statistically compared between good and poor recovery groups, and several machine learning models were trained with different combinations of features to predict patient outcome.

**Results:**

A threshold between lower and upper severe disability (GOS-E = 4) optimized classification of recovery. Criticality-related and spectral EEG features differed significantly between good and poor recovery groups, and EEG features predicted patient recovery above and beyond patient demographics, with a maximal predictive accuracy of 87% and AUC of 0.92. Patients with good outcomes exhibited greater EEG complexity, entropy and fractal patterns compared to those with poor outcomes.

**Conclusion:**

This study demonstrates the prognostic potential of EEG spectral and criticality-related features in predicting outcomes for sedated PICU patients.

## 1 Introduction

Predicting the capacity for recovery following severe injury underpins some of the most important decisions in the pediatric intensive care unit (PICU). This task remains a major challenge in children who are unresponsive following a critical illness due to the lack of reliable behavior-independent prognostic markers. Recent guidelines have recommended the integration of multimodal assessment, including neurophysiological data, to provide more accurate insights for unresponsive pediatric patients (Boerwinkle et al., 2023). Electroencephalography (EEG) is commonly deployed at the bedside in the PICU; it has been used to assess levels of consciousness (Sitt et al., 2014; Kesić and Spasić, 2016; Walter and Hinterberger, 2022a; Liu et al., 2023) and depth of anesthesia in healthy children (Puglia et al., 2021), as well as in healthy (Liang et al., 2015; Schartner et al., 2015; Colombo et al., 2019) and brain-injured adults (Maschke et al., 2023; Liang et al., 2020; Ballanti et al., 2022; O’Donnell et al., 2021). Although EEG features and characteristics vary with age (Li et al., 2020; Biggs et al., 2022; Purdon et al., 2015), they remain promising candidates for predicting recovery of consciousness in unresponsive pediatric patients.

A promising mechanistic framework for interpreting EEG during impaired consciousness is the critical brain hypothesis (Chialvo, 2010; O’Byrne and Jerbi, 2022). According to the critical brain hypothesis, neural systems optimally operate near a boundary, at the edge of order and chaos, where the brain’s capacity for information processing, flexibility, and adaptability is maximized (Shew and Plenz, 2013; O’Byrne and Jerbi, 2022). The electrophysiological patterns supporting consciousness have been proposed to emerge at this boundary between stability and flexibility that enables effective information integration and segregation required for conscious processing (Toker et al., 2022). Accordingly, deviations from this regime can be induced pathologically (e.g., diffuse injury) or pharmacologically (e.g., anesthesia), yielding overly ordered or disordered dynamics that impair cognitive function and disrupt consciousness (Gervais et al., 2023; Hengen and Shew, 2025). This framework provides a compelling approach for assessing the capacity for the recovery of consciousness in the absence of behavioral responsiveness (Walter and Hinterberger, 2022b; Zimmern, 2020). In the PICU, sedation provides a clinically routine pharmacological perturbation that suppresses excitability and narrows the brain’s dynamical repertoire. Therefore, EEG recorded under sedation can be interpreted as reflecting the residual capacity of injured neural systems to maintain critical dynamics which facilitate adaptability; a neurophysiological “reserve” relevant to recovery.

Brain criticality can be measured through a diverse set of perspectives and methods (O’Byrne and Jerbi, 2022). However, most direct measures of criticality (e.g., avalanche criticality, edge-of-chaos criticality) are highly sensitive to noise, and often incompatible with the signal-to-noise constraints of EEG recorded at the bedside in the PICU. Instead, we turn to a set of criticality-related measures that are more tolerant to the signal quality of clinical EEG: entropy, fractality, and complexity. Entropy features reflect signal predictability; fractal features capture scale-free patterns; and complexity features describe structured patterns and signal stability. All three families of measures have shown strong association to the brain’s underlying criticality (Werner, 2010). Within the critical brain framework, greater entropy, scale-free patterns, and richer complexity in EEG signals are theorized to reflect neural dynamics closer to a critical state optimizing flexible information processing, dynamic range, and adaptability. By measuring these signatures in sedated pediatric brain-injured patients, we aim to capture the neurophysiological markers of residual dynamics that may predict later functional outcome.

In this study, we aimed to assess the association between the brain criticality of sedated children in the PICU and their outcomes. We hypothesized that deviations from a critical state reflected by entropy, fractal and complexity features of the EEG would predict capacity for recovery, with participants whose EEG was closer to a critical state demonstrating better functional outcomes.

### 2 Materials and methods

#### 2.1 Study design

We conducted a multi-center, retrospective cohort observational study with patients admitted to the PICU at McMaster Children’s Hospital and Alberta Children’s Hospital in Canada. This study was approved by the Hamilton Integrated Research Ethics Board under the project 13848-C and University of Calgary Research Ethics Board under REB23-0116 and REB20-1913.

### 2.2 Participants

We reviewed all clinical EEGs collected between 2014 and 2023 for McMaster Children’s Hospital and between 2019 and 2024 for Alberta Children’s Hospital for patients that met the following inclusion criteria: had a severe brain functional impairment; were between 5 and 18 years of age; were receiving sedation with a GABAergic anesthetic (propofol or midazolam); and had a functional outcome measurement at approximately 3 months post-ICU discharge. Patients who received GABAergic sedatives in combination with dexmedetomidine and/or opioid analgesic such as fentanyl or hydromorphone were also included. The lower age limit of 5 years was set to ensure the consolidation of the posterior dominant rhythm in the participant’s EEG. Inhibitory anesthetics were included as they are typically used in the standard of care in the PICU. A total of *n* = 32 participants were identified (mean age: 11.34 ± 3.19 years; 14 females; 28 from McMaster Children’s Hospital). Participants were categorized according to their etiology: acute seizures (28%), acute brain injuries (22%), and systemic illness (50%). Acute seizures included acute exacerbation and worsening seizures in patients with epilepsy, febrile seizures, refractory status epilepticus and syndromes with a high propensity of seizures (Dravet syndrome and Sturge-Weber syndrome). Acute brain injuries included primary stroke (hemorrhagic and ischemic) and TBI (including concomitant diffuse axonal injury, intracranial hemorrhage). Systemic disorders included infections (viral encephalitis and *Staphylococcus aureus*), metabolic crisis (diabetic ketoacidosis and acute kidney injury), and anoxia (secondary to cardiac arrest, strangulation, and submersion).

EEG recordings were clipped during steady-state anesthetic infusions to a length varying between 5 and 30 min. To minimize acute medication related confounding, analyzed EEG segments were selected when the primary sedative regimen was stable, and no additional medication expected to alter EEG activity was administered. A detailed summary of sedative type, dose, and relevant demographic information for each analyzed sample is provided in Supplementary Table 6. EEG recordings from the seizure group were taken during interictal periods such that no seizure activity or post-ictal slowing was included in the analysis. Several participants had multiple steady-state periods, which were all included, as they reflected variability in dosage, sedatives, and points along the recovery trajectory.

### 2.3 EEG preprocessing

EEG was recorded from 26 channels at McMaster Children’s Hospital and from 20 channels at Alberta Children’s Hospital, with placement following the standard international 10-20 system. Clipped EEG recordings were preprocessed using an automated pipeline in MNE-Python (Gramfort et al., 2013), along with the Autoreject (Jas et al., 2017) package. Electrooculogram (EOG) and electrocardiogram (EOG) channels were identified and excluded. EEG signals were down-sampled to 250 Hz, bandpass filtered between 0.5 and 45 Hz, and notch filtered at 60 Hz to reduce power line noise. The data were then re-referenced to the mastoid electrodes. Independent Component Analysis (ICA) was conducted to exclude EOG and ECG artifacts. The recordings were then segmented into 10-s epochs. Bad epochs were identified and interpolated using Autoreject with a 10-fold cross-validation procedure.

### 2.4 EEG feature extraction and analysis

We calculated EEG features from three criticality-related categories: entropy, fractality and complexity. Certain features require phase-space reconstruction of the time series. Reconstruction parameters, including delay and embedding dimension, and all subsequent features were computed using the Neurokit package (Makowski et al., 2021). Delays were calculated using the mutual information approach (Fraser and Swinney, 1986), and embedding dimensions were determined using the average false neighbors’ method (Cao, 1997).

*Entropy features*: We used three entropy features to characterize EEG predictability/uncertainty: (1) permutation entropy, which quantifies the complexity of the time series through the log probabilities of ordinal patterns in phase space; (2) conditional weighted permutation entropy, which corrects permutation entropy by incorporating conditional probabilities and amplitude weightings; and (3) sample entropy, which measures the natural logarithm of the ratio of vector similarities in the phase space, reflecting the time series predictability.

*Fractal features:* We used four fractal features to assess scale-free dynamics and long-range temporal correlations within the EEG. (1) The Higuchi fractal dimension captures the complexity of the time series across temporal scales. (2) The Hurst Exponent quantifies long-range temporal correlations and was estimated using Detrended Fluctuation Analysis (DFA). For DFA, longer time series are required to estimate long-range temporal correlation reliably. After preprocessing and rejection of bad epochs, retained clean 10-s epochs were concatenated into 90-s time series for DFA estimation. This concatenation step was used only for DFA and was not applied to the other EEG features. The concatenated signal was band-passed filtered in the alpha frequency range, and the amplitude envelope was extracted using the Hilbert transform. We then used DFA on the alpha-band amplitude envelope to estimate the slope of the variance across progressively larger window sizes, capturing the similarity of the signal across temporal scales. Additionally, we calculated (3) the spectral slope and (4) the spectral offset using the FOOOF software package (Donoghue et al., 2020), which characterizes the power-law decay of the EEG power spectrum, reflecting patterns across multiple timescales.

*Complexity features:* We used two complexity features to assess temporal patterns and signal stability. (1) Lempel-Ziv Complexity (LZC) quantifies structured patterns of time series. We mean binarized the EEG time series, calculated LZC on the binary sequence and normalized by the signal length. (2) Lyapunov Exponent captures the stability of the signal’s trajectory in phase space, quantifying the sensitivity to initial conditions. We calculated this feature using the Rosenstein method (Rosenstein et al., 1993).

Feature selection was hypothesis-driven and limited to reduce redundancy and overfitting in this small retrospective cohort, while preserving measures that capture complementary aspects of entropy, fractality and complexity. In addition, as clinical EEG is traditionally characterized through power-band analysis, we also calculated five spectral features to enable comparison of the criticality-related features to these more common measures.

*Spectral features:* We calculated spectral power using the Welch method from MNE Python, and estimated power for each of the canonical frequency bands: delta (1–4 Hz), theta (4–8 Hz), alpha (8–13 Hz), beta (13–30 Hz), and gamma (30–45 Hz). Absolute power for each band was calculated by integrating the area under the curve using Simpson’s rule (Scipy library) (Virtanen et al., 2020) within each frequency range.

### 2.5 Outcome measurements

Patient outcomes were assessed using the Glasgow Outcome Scale-Extended (GOS-E) three months post-injury (Wilson et al., 2021). The GOS-E is an ordinal measure that categorizes functional recovery from 1 (death) to 8 (upper good recovery). To binarize patients into “good” and “poor” recovery groups, an odds ratio analysis was conducted to identify an optimal GOS-E cut-off threshold.

### 2.6 Statistical analysis

#### 2.6.1 Feature aggregation

Each EEG feature was aggregated across all epochs and channels for each patient to obtain a representative measure. The normality of each feature distribution for each recording was assessed using the Shapiro-Wilk test. Most recordings failed the test; thus, we used the median to characterize all aggregate features.

#### 2.6.2 Group comparisons

The aggregated features did not meet the assumptions required for parametric analysis (Supplementary Figure 1) and were therefore analyzed using non-parametric tests. Group comparisons between good and poor recovery patients were performed using Mann-Whitney U tests for each feature. To correct for multiple comparisons, all *p*-values were adjusted using the Bonferroni correction.

To assess whether differences in available EEG data could bias outcome-related analyses, we quantified the percentage of EEG data retained after preprocessing and quality control for each analyzed recording and compared this between functional outcome groups using a Mann-Whitney U test.

#### 2.6.3 Ordinal ratio analysis

An ordinal ratio analysis was performed to identify the optimal GOS-E threshold for classifying recovery while accounting for group heterogeneity. Odds ratios and 95% confidence intervals were calculated for various thresholds, with values closer to 1 indicating minimal group imbalance. Mann-Whitney U tests were used to assess the statistical significance of feature differences at each threshold. The optimal threshold was identified by the most significant *p*-values across features, balancing statistical robustness and clinical relevance.

### 2.7 Machine learning analysis

We trained several machine learning models to predict patient outcome from various combinations of the nine criticality-related EEG features, the five spectral EEG features, and the demographic features (age, sex, etiology). All models were tested using a 5-fold stratified cross validation. The primary model was a logistic regression model that included all EEG and demographic features. Categorical variables, such as sex and etiology, were one-hot encoded, while age was treated as a continuous variable. Features were standardized prior to model training to maximize comparability across predictors. Additional models were trained to evaluate the contribution of only the demographic features, and only the EEG features.

Three classifiers were evaluated with their default parameters using scikit-learn (version: 1.5.2.) (Pedregosa et al., 2011): logistic regression, linear discriminant analysis (LDA) and support vector machines (SVM) with probability estimation. Model performance was assessed using F1-score, and area under the receiver operating characteristic (ROC) curve (AUC). To address the class imbalance inherent in the dataset, bootstrapping was performed on the logistic regression model using the full feature set. Feature importance and confusion matrices were averaged across the bootstrap iterations to quantify the contributions of each predictor and to assess the model’s overall classification performance, including sensitivity and specificity.

## 3 Results

### 3.1 A threshold between lower and upper severe disability optimizes classification of recovery

We selected the optimal threshold to distinguish good and poor recovery through a combination of ordinal ratio analysis and statistical significance testing. Ordinal ratio analysis evaluated group balance across varying GOS-E thresholds, with odds ratios and confidence intervals presented for each threshold (Supplementary Figure 2). To validate this threshold, Mann-Whitney U tests were conducted on EEG features across thresholds. The mean optimal threshold across all features was 4.07 (SD = 1.79). Considering the similarity in odds ratios across thresholds of 4, 5 and 6, the statistical analysis supported the use of GOS-E ≥ 4 as the recovery threshold. This threshold provides a compromise between group balance and feature differentiation, with a clinically relevant threshold representing functional independence in recovered patients. For all subsequent analyses, GOS-E scores of ≥ 4 were classified as “good recovery,” while scores < 4 were classified as “poor recovery.”

### 3.2 Criticality-related and spectral EEG features differ between good and poor recovery groups

Group comparisons between good recovery (*n* = 22; 117 files) and poor recovery (*n* = 10; 58 files) were conducted using Mann-Whitney U tests across all EEG features and corrected for multiple comparisons. Seven of the fourteen features were significantly different (*p* < 0.05) between good and poor recovery groups, indicating distinct neural dynamics associated with recovery (Table 1).

**TABLE 1:** Group comparisons of EEG features between patients with good recovery and poor recovery.

Feature	U Statistic	P-Value	Median (good recovery)	Median (poor recovery)
Lempel-Ziv complexity	5253.500	< 0.0001	0.23	0.13
DFA alpha	4687.000	0.0005	0.66	0.59
Higuchi fractal dimension	4523.000	0.0048	1.91	1.90
Sample entropy	4151.000	0.2288	0.12	0.09
Permutation entropy	4093.000	0.3725	0.87	0.85
Spectral slope offset	3014.000	1.00	−0.43	−0.34
Conditional weighted permutation entropy	2896.000	1.00	0.08	0.09
Lyapunov exponent	2768.000	0.6687	0.00	0.00
Spectral slope	2071.000	0.0004	2.30	2.81
Absolute beta power	5645.000	< 0.0001	0.02	0.00
Absolute alpha power	5347.000	< 0.0001	0.02	0.00
Absolute gamma power	4613.000	0.0016	0.00	0.00
Absolute theta power	4231.000	0.1111	0.04	0.03
Absolute delta power	2981.000	1.00	0.36	0.40

Mann-Whitney U tests (with Bonferroni correction for multiple comparisons) for group comparisons of 14 EEG features between good recovery (GOS-E ≥ 4) and poor recovery (GOS-E < 4) patients. Including U statistics, *p*-values, and median values for each feature across both groups.

To determine whether these group differences could be explained by unequal amounts of usable EEG data, we compared the percentage of EEG data retained after preprocessing and quality control between outcome groups. Retained EEG data did not differ significantly between good and poor recovery groups (*U* = 3511.000, *p* = 0.71; Supplementary Figure 4), suggesting that outcome-related differences in EEG features were unlikely to be driven by differences in recording length or data retention.

Three of nine criticality-related EEG features were significantly higher in good recovery patients (Lempel-Ziv Complexity (*U* = 5253.500, *p* < 0.001); DFA alpha (*U* = 4687.000, *p* < 0.001); Higuchi fractal dimension (*U* = 4523.000, *p* = 0.004)), while spectral slope (*U* = 2071.000, *p* < 0.001) was significantly lower in the good recovery group. Three of five spectral EEG features were also significantly different between good and poor recovery patients, with absolute beta power (*U* = 5645.000, *p* < 0.001), absolute alpha power (*U* = 5347.000, *p* < 0.001), and absolute gamma power (*U* = 4613.000, *p* = 0.002) higher in the good recovery group.

**FIGURE 1:**
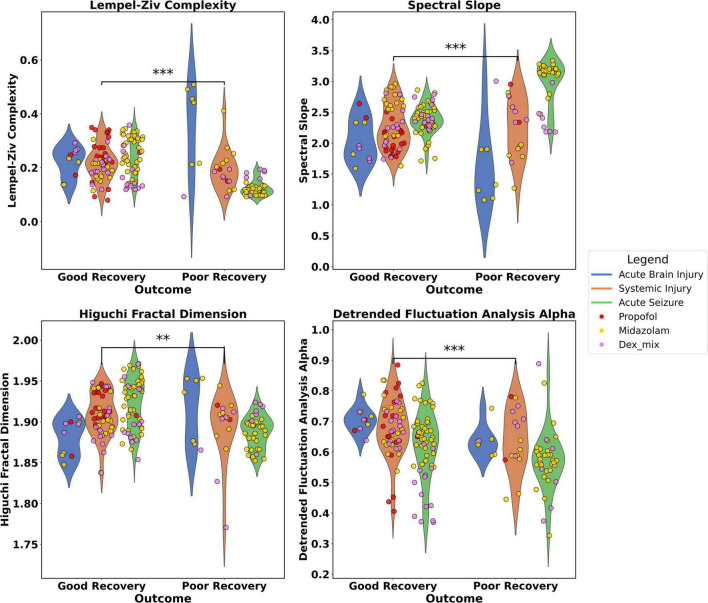
EEG feature differences between good recovery (GOS-E >=4) and poor recovery groups for Lempel-Ziv Complexity, Spectral Slope, Higuchi Fractal Dimension, and DFA Alpha. Significance levels from Mann-Whitney U tests (with Bonferroni correction for multiple comparisons) are indicated (**p < 0.01, ***p < 0.001). For visualization, groups are split by etiologies represented as violin plots: acute brain injury (blue), systemic injury (orange), acute seizure (green). Within each etiological subgroup, the scatter plots represent sedative agent: propofol (red), midazolam (yellow), dexmedetomidine with a GABAergic sedative (purple).

Group differences in four representative criticality-related EEG features—Lempel-Ziv Complexity, spectral slope, Higuchi fractal dimension, DFA alpha—are presented in Figure 1, stratified by etiology and sedative type (see Supplementary Figure 3 for the remaining significant features). These results demonstrate that criticality-related and spectral EEG features distinguish neural dynamics between recovery groups.

We conducted a *post-hoc* analysis to evaluate the effect of different sedation types on the EEG features’ separability between recovery groups. We performed Mann-Whitney U tests for each EEG feature within each sedative subgroup (midazolam, propofol, dexmedetomidine with a GABAergic sedative). EEG features differentiated recovery groups predominantly under midazolam anesthesia (8/14 significant features), while propofol and dexmedetomidine induced no significant changes in criticality-related and spectral EEG to distinguish between groups (Supplementary Table 3).

We then conducted a second *post-hoc* analysis to evaluate the effect of etiology on the EEG features’ separability between recovery groups. We performed Mann-Whitney U tests for each EEG feature within each etiology subgroup (acute brain injury, systemic injury, acute seizure). EEG features were significantly different between recovery groups only in acute seizure (9/14 significant features), with acute brain injury and systemic injury yielding no differences to distinguish groups (Supplementary Table 4). These results indicate that outcome-related EEG feature differences were not uniformly expressed across etiologies but were most robust in the acute seizure subgroup.

### 3.3 EEG spectral and criticality-related features predict recovery above and beyond patient demographics

We evaluated the prognostic value of demographic, EEG spectral and EEG criticality-related features using three machine learning classifiers. The logistic regression model predicted good versus poor recovery with an accuracy of 81% and an AUC of 0.85. This model achieved a precision of 74%, recall of 67%, and F1-score of 70% for the poor recovery class and a precision of 84%, recall of 88%, and F1-score of 86% for the good recovery class. Weighted averages were consistent at 81% for precision, recall, and F1-score, accounting for the class distribution, indicating a strong overall performance with higher sensitivity for the recovered group.

Using the same feature set, the LDA model achieved an accuracy of 82% and an AUC of 0.82, with an F1-score of 70% for the poor recovery class and 87% for the good recovery class. The SVM model achieved the highest accuracy of 87% and AUC of 0.92, with significantly improved precision of 97% and F1-score of 76% for the non-recovered class, while maintaining strong performance for the recovered class (F1-score: 91%). The weighted-average F1-score for SVM of 86% outperformed that of LDA, 82%, and logistic regression, 81%, highlighting its robustness across both classes.

**FIGURE 2:**
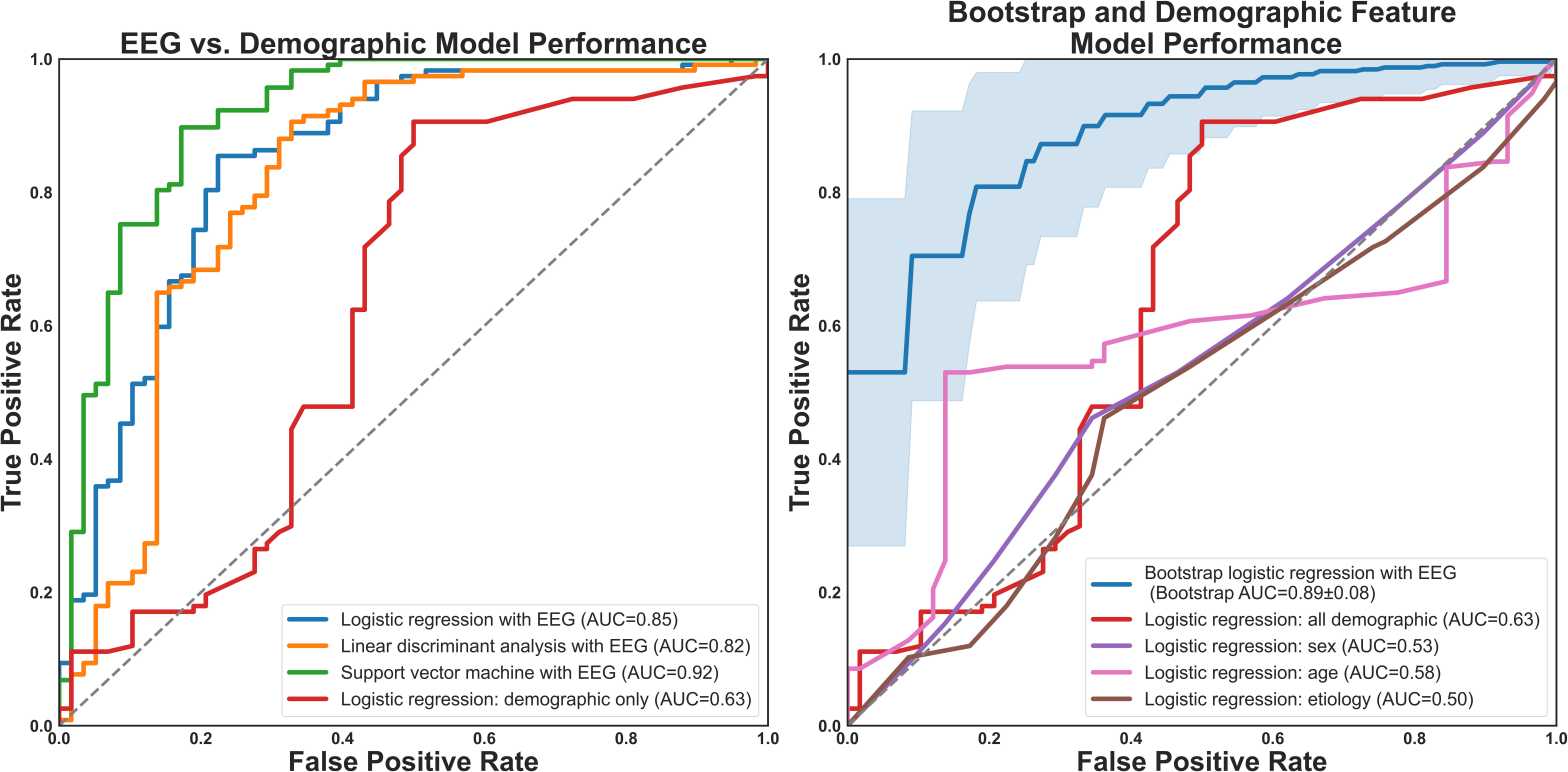
ROC characterization of model performance. Left: Comparison of models trained on EEG and demographic features versus demographic features alone (red). Three models were trained with spectral and criticality-related EEG features added to demographic features: logistic regression (blue), LDA (orange), and SVM (green). Right: Control model comparisons. Bootstrapped ROC performance for the logistic regression with EEG and demographic features, with the mean ROC curve (solid line) and variability across iterations (shaded region) in blue. ROC characterization of logistic regression models trained on all demographic features (red), sex (purple), age (pink) and etiology (brown).

We then evaluated the added value of the EEG features above and beyond demographic factors to predict participant outcome. Logistic regression models trained on demographic features (participant age, sex, and etiology) alone achieved 77% accuracy and an AUC of 0.63, with the good recovery group achieving an F1-score of 84% and the poor recovery group 59%. Models trained on the individual demographic features performed worse, with overall accuracies of 66–67%. All models trained with EEG features consistently outperformed those trained without them (Figure 2). Bootstrap analysis confirmed the stability and robustness of the models’ performance despite the class imbalance between good and poor recovery groups (Supplementary Table 5).

**FIGURE 3:**
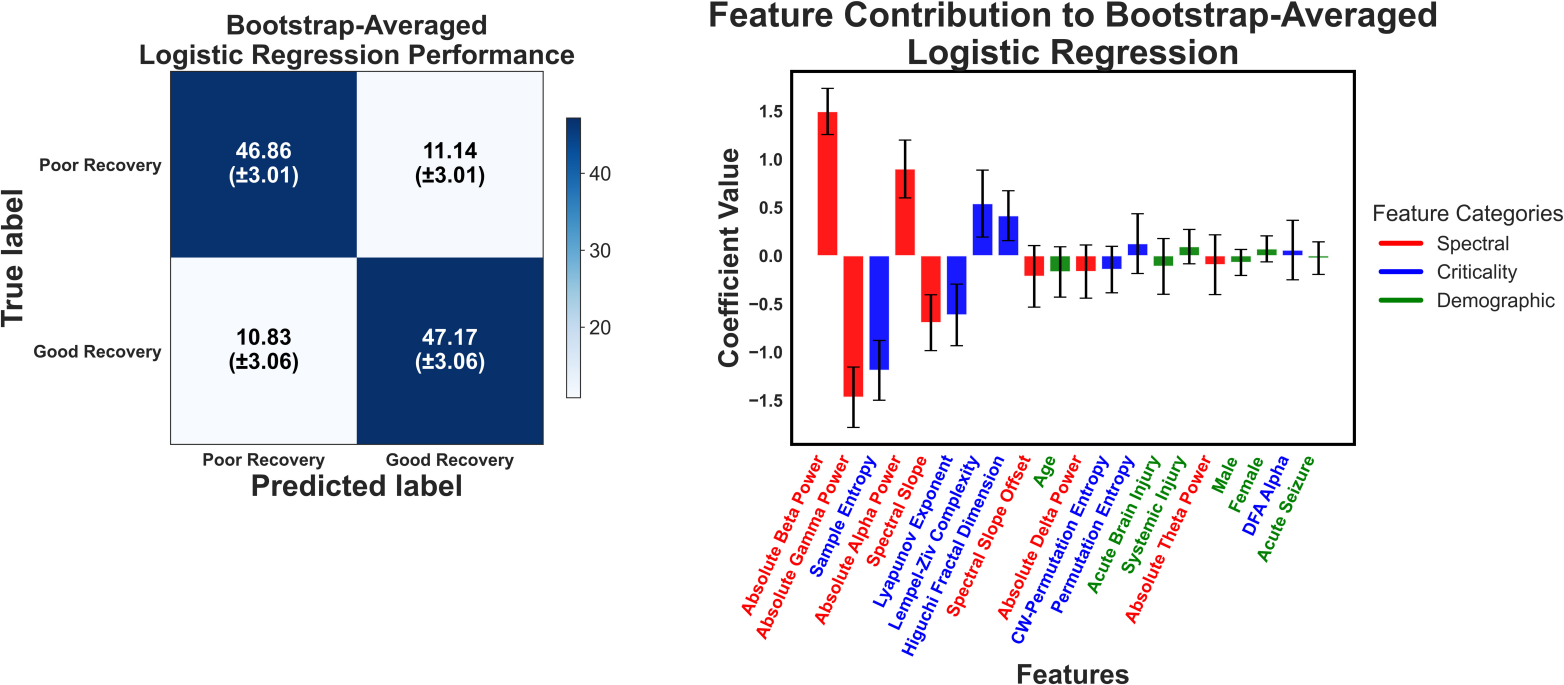
Relative feature contributions to the bootstrap-averaged logistic regression prediction of patient outcome. Left: Confusion matrix for the logistic regression model trained on all EEG and demographic features, averaged across bootstrapped iterations with standard deviation in parentheses, to account for class imbalance between good and poor recovery groups. Right: Contribution of each feature to model performance assessed by logistic regression coefficients averaged across bootstrap iterations (error bars represent standard deviation). Features are categorized as demographic (green), EEG spectral (red), and EEG criticality-related (blue).

Finally, we assessed the relative contributions of each of the 17 features to the predictive performance of the model. Although the SVM achieved the highest AUC of 0.92, this model combined features in a non-linear manner; thus, we selected the logistic regression for the analysis, as it had the next highest model performance and maximal interpretability of feature contribution. To address the class imbalance between good and poor recovery groups, we assessed model performance and feature importance on the bootstrapped model. The average bootstrapped model had high sensitivity for the good recovery group, correctly classifying 47.17 (± 3.06) of 58 cases with good recovery; and high specificity for the poor recovery group, correctly classifying 46.86 (± 3.01) of 58 cases (Figure 3, left). Feature importance was assessed by averaging logistic regression coefficients across bootstrapped iterations. EEG spectral features, such as absolute beta power and absolute gamma power, contributed the most to the model’s predictions, followed by criticality features, such as sample entropy, spectral slope, Lyapunov exponent and Lempel-Ziv complexity (Figure 3, right). Demographic features had comparatively lower contributions to the model’s performance.

## 4 Discussion

In this study, we investigated the prognostic potential of EEG spectral and criticality-related features in sedated children admitted to the PICU. Grounded in the critical brain hypothesis, when neural dynamics operate near a critical regime that balances stability and flexibility, maximizes adaptability and supports consciousness processing. Sedation acts as a pharmacological suppressive perturbation that narrows the available dynamical repertoire of the brain. The EEG observed under sedation therefore reflects the brain’s residual capacity to maintain critical dynamics and sustain functional recovery. We quantified critical dynamics through clinically tractable EEG features and tested whether they encode prognostic information for recovery of functional outcome in the sedated brain-injured pediatric population. Using an ordinal ratio analysis, we established a threshold of GOS-E =4 to distinguish good versus poor recovery outcomes; this threshold also has the benefit of being a clinically relevant assessment threshold suggestive of functional independence. We demonstrated that three of five EEG spectral features and four of nine EEG criticality-related features were significantly different between good and poor recovery groups, with participants whose EEG was closer to a critical state having better functional outcomes. A machine learning model trained on patient demographic and EEG spectral and criticality-related features achieved a classification accuracy of 87%, with the EEG providing significantly more predictive power than the demographic features. Altogether, our results highlight the prognostic power of EEG patterns as measures of brain dynamics for predicting recovery in the absence of clinical responsiveness in pediatric intensive care.

Building on previous research, which demonstrated changes in pediatric EEG complexity under anesthesia (Puglia et al., 2021), our findings reveal the valuable role of criticality features in distinguishing recovery outcomes. Three of the criticality-related features that we selected, spectral slope, DFA, and Higuchi fractal dimension, characterize temporal fractal patterns that support flexible neural information processing across temporal scales, rather than at a dominate frequency of activity. Flatter spectral slope and larger DFA and Higuchi fractal dimension values suggest cross-frequency activity and broader temporal scale integration. These dynamics were observed in patients that had a good recovery, emphasizing that the proximity to criticality supports more flexible and dynamic brain activity, facilitating adaptive neural processing following impairment, injury, or sedation. Within the critical brain hypothesis, this pattern is consistent with a system that retains access to a broader dynamical range rather than collapsing into overly ordered or disordered dynamics that can constrain information propagation and adaptability needed for the recovery of functional outcomes.

Two criticality-related features, permutation and sample entropy, characterized the EEG signal’s predictability; consistent with the critical brain hypothesis, the good recovery patients had higher entropy brain dynamics. Similarly, greater temporal pattern variability, captured by Lempel-Ziv complexity, characterized the good recovery group. Taken together, the convergence of flatter aperiodic structure, stronger scale-free temporal organization and greater richness in electrophysiology support the interpretation that better outcome patients exhibit dynamics that are neither too rigid nor too random. These findings underscore the importance of modeling the brain as a complex adaptive system to evaluate critical dynamics that may help predict recovery. It is important to note that all samples of clinical EEG analyzed in our study were collected while the participants were under varying doses of GABAergic sedation, and segments were selected to minimize overlap with additional medications expected to alter EEG activity (i.e., changes in beta activity, slowing, burst suppression and spindle like patterns). By suppressing brain activity, these sedatives reveal shifts in the excitation-inhibition balance—a key component of criticality (Liang et al., 2025). Therefore, the EEG signal measured under sedation is not only a suppression of neural activity, rather it is a window into the brain’s residual critical dynamics and self-organizing capacity. Patients who maintain signatures of criticality under pharmacological suppression appear to retain sufficient neurophysiological capacity to support functional recovery. Sedation can be interpreted as a controlled perturbation that probes how resiliently the injured brain can preserve structured variability under reduced excitability.

The use of clinical EEG for prognostication is a well-established practice in critical care. Traditionally, clinical EEG analysis has focused on waveform characteristics and spectral features, such as the presence or absence of a posterior dominant rhythm (Labonte et al., 2023). In pediatric neurocritical care, EEG background abnormalities such as low voltage, flat tracing, delayed recovery of continuous background activity, diminished signal complexity, reduced variability, and increased monotony have also been associated with severe encephalopathy and unfavorable outcomes (Proietti et al., 2024; Hess et al., 2026). Quantitative EEG provides objective and scalable biomarkers of brain dysfunction, including measures of amplitude, spectral power, entropy and fractal structure. This study advances this practice by situating EEG analysis within the theoretical framework of brain criticality, moving beyond descriptive spectral characterization toward mechanistically grounded framework of neural dynamics. Our results affirm the prognostic value of EEG spectral features, which emerge as the top contributors to the predictive power in a logistic regression model of recovery. The addition of EEG criticality-related features to demographic and spectral features improves the classification of recovery by considering the underlying dynamics of the system. In this context, changes in EEG criticality-related features may reflect impaired cortical-subcortical coupling, reduced network differentiation, and diffuse brain dysfunction, whereas preserved entropy, fractality, and complexity under sedation may indicate residual capacity for flexible neural information processing. Though criticality-related features have shown promise in predicting recovery of consciousness in adult populations, this is the first study to demonstrate the prognostic potential of these metrics in pediatric patients in intensive care (Maschke et al., 2023). Given the heterogeneity of PICU patients, which differ based on etiology, sedation (agents, duration, depth), and patient-specific factors, EEG criticality features offer a means to differentiate observable clinical phenotypes from underlying neurophysiological endotypes—differential biomechanical mechanisms resulting in a similar clinical presentation of unresponsiveness (Azad et al., 2022). Importantly, outcome-related EEG differences were not uniform across etiologies. In stratified analyses, EEG features most robustly distinguished good and poor recovery in the acute seizure subgroup, whereas acute brain injury and systemic illness showed weaker separability. This suggests that the prognostic value of sedation-related EEG dynamics may depend on clinical context, with acute seizure patients potentially showing a more consistent relationship between preserved critical dynamics and recovery potential. These findings require validation in larger etiology-specific prospective cohorts. This approach enables a more nuanced understanding of patient trajectories and may support the development of additional monitoring and prognostic tools in pediatric neurocritical care. Specifically, criticality-based endotype differentiation offers theoretically motivated measurements such that patients with similar clinical presentations may be meaningfully characterized based on underlying organization of neural dynamics. This conceptual shift represents the potential of personalized approaches to neurocritical care.

This study has several notable strengths. First, the participants included in this study were extremely heterogenous, with large variations in age, etiology, sedation protocols, and course in PICU, therefore being representative of the diversity of the PICU population. The classifiers were able to predict patient recovery with over 80% accuracy across this dataset, indicating the broad applicability of the measures across patient categories and the enormous translational potential of our approach. Second, the dataset was sourced from two different PICUs with two different clinical EEG systems, again highlighting the translational potential and broad applicability of our approach. By collecting retrospective data across two sites, the findings highlight the ecological validity and generalizability of using EEG-based prognostic tools in diverse clinical settings. Third, EEG was collected when participants were under sedation. This approach aligns with standard clinical protocols, takes advantage of the EEG monitoring in the ICU, and does not require the clinical team to change their standard of care, further supporting its practical applicability for real-world decision-making. Together, these strengths position the present work as a contribution to the critical brain hypothesis by demonstrating criticality-related signatures of EEG signals can be extracted and applied across real world clinical contexts.

The results of this study also need to be considered in light of several limitations. First, our approach does not estimate criticality directly (e.g., via avalanche scaling exponents or edge-of-chaos statistics) but instead uses proxy signatures that are theoretically related to critical dynamics and are more robust to the readily available bedside PICU EEG. Second, multiple recordings were included from individual participants to reflect different stages in their recovery trajectory and varying dosages of sedatives. These samples cannot be considered independent, but this approach provided an opportunity to capture the dynamic progression of brain recovery in the PICU and to capture the non-linear dose-dependent responses to sedation. Third, we used whole brain-averaged features, which are well-suited for clinical EEG; however, this method overlooks spatial differences in brain activity which may be markers of impairment leading to future functional impact. Future work examining these spatial patterns could provide further insights into prediction of recovery and clarify whether criticality-related signatures reflect global dynamics or specific network level heterogeneity. Additionally, DFA required concatenation of retained clean epochs to obtain sufficiently long time series for estimating long-range temporal correlations. Although this procedure was applied only to DFA and retained-data percentage did not differ between outcome groups, concatenation may alter temporal structure at epoch boundaries. Fourth, the small sample size for certain subgroups of anesthetics and etiologies limits statistical power and generalizability. This small sample size also reflects the retrospective selection process and may have introduced selection bias by excluding milder patients who did not require EEG monitoring, patients without follow-up outcome assessment, and EEG recordings that contained seizures, post-ictal slowing, burst-suppression, or medication changes likely to affect EEG activity. The non-significant results for the propofol and dexmedetomidine sub-analyses are likely due to lack of power, and future prospective studies are required to assess the prognostic effect of brain criticality under these anesthetics conditions. In addition, EEG features in the PICU may reflect not only the primary injury but also secondary injury processes, treatment effects, sedation depth, and evolving recovery. These factors may mask or modify primary injury-related EEG patterns. Therefore, future prospective studies should standardize EEG timing relative to injury, treatment exposure and recovery stage, particularly during the early post-injury period when injury-related EEG changes may be most evident. Fifth, our study did not consider age and developmental variability, which both play a significant role in recovery trajectories. Younger patients exhibit greater neural plasticity, which influences recovery potential (Johnston, 2009). Future studies could stratify outcomes by developmental stages or etiological differences, providing a more nuanced understanding of age-related and injury-related differences in brain dynamics and recovery potential. This stratification could identify distinct endotypes, where specific age and injury profiles reveal unique neurophysiological mechanisms underlying recovery. Sixth, the small size of our dataset required us to binarize into “good” and “poor” recovery classes. With larger cohort sizes, employing a sliding dichotomy approach could allow for dynamic adjustment of GOS-E thresholds based on patient-specific characteristics, enhancing the predictive power of EEG features for recovery outcomes. Finally, the dataset exhibited a significant class imbalance, with poor recovery patients being underrepresented. This imbalance could bias the model toward the majority class, reducing the ability to accurately predict outcomes for patients that do poorly. To address this, we employed bootstrapping techniques, which involved resampling the data with replacement to create balanced datasets for training. Bootstrapping enhanced the robustness and reliability of the predictive models with a consistent accuracy of 81% (± 4.3%). These findings demonstrate that, while diverse EEG features show strong potential for predicting recovery, expanding the dataset with balanced groups could enhance the model’s performance providing greater utility for clinical decision-making.

## 5 Conclusion

This study demonstrates the prognostic utility of EEG features in predicting recovery outcomes for patients in the PICU under sedation. Among the 14 EEG features analyzed, 7 were statistically significant in distinguishing recovery determined by functional outcome. EEG criticality-related features provided valuable insights into brain dynamics and capacity for recovery. Machine learning models leveraging these features achieved accuracies exceeding 80% across classifiers, highlighting their effectiveness in outcome prediction. These findings support the translational relevance of the critical brain hypothesis by demonstrating that clinically tractable EEG criticality-related features (entropy, fractality, and complexity) are associated with prognostic information related with functional outcomes. Bringing criticality motivated analysis into real world neurocritical care with the integration of these temporal EEG features may improve clinical decision-making, offering a valuable tool for outcome assessment in pediatric critical care settings.

## Data Availability

The data analyzed in this study is subject to the following licenses/restrictions: This is patient data and cannot be publicly shared. Requests to access these datasets should be directed to stefanie.blain-moraes@ubc.ca.
