# Supplementary material for: EEG criticality as a prognostic tool for functional outcomes in sedated pediatric intensive care patients
Source: Front Comput Neurosci. 2026 Jul 8;20:1831476. doi: 10.3389/fncom.2026.1831476 (PMC13388815; doi:10.3389/fncom.2026.1831476)
Supplement: Supplementary file 1 [file Data_Sheet_1.docx]

**Supplementary Material for Manuscript:** EEG Criticality as a Prognostic Tool for Functional Outcomes i­­­n Sedated Pediatric Intensive Care Patients

**Figure S1**


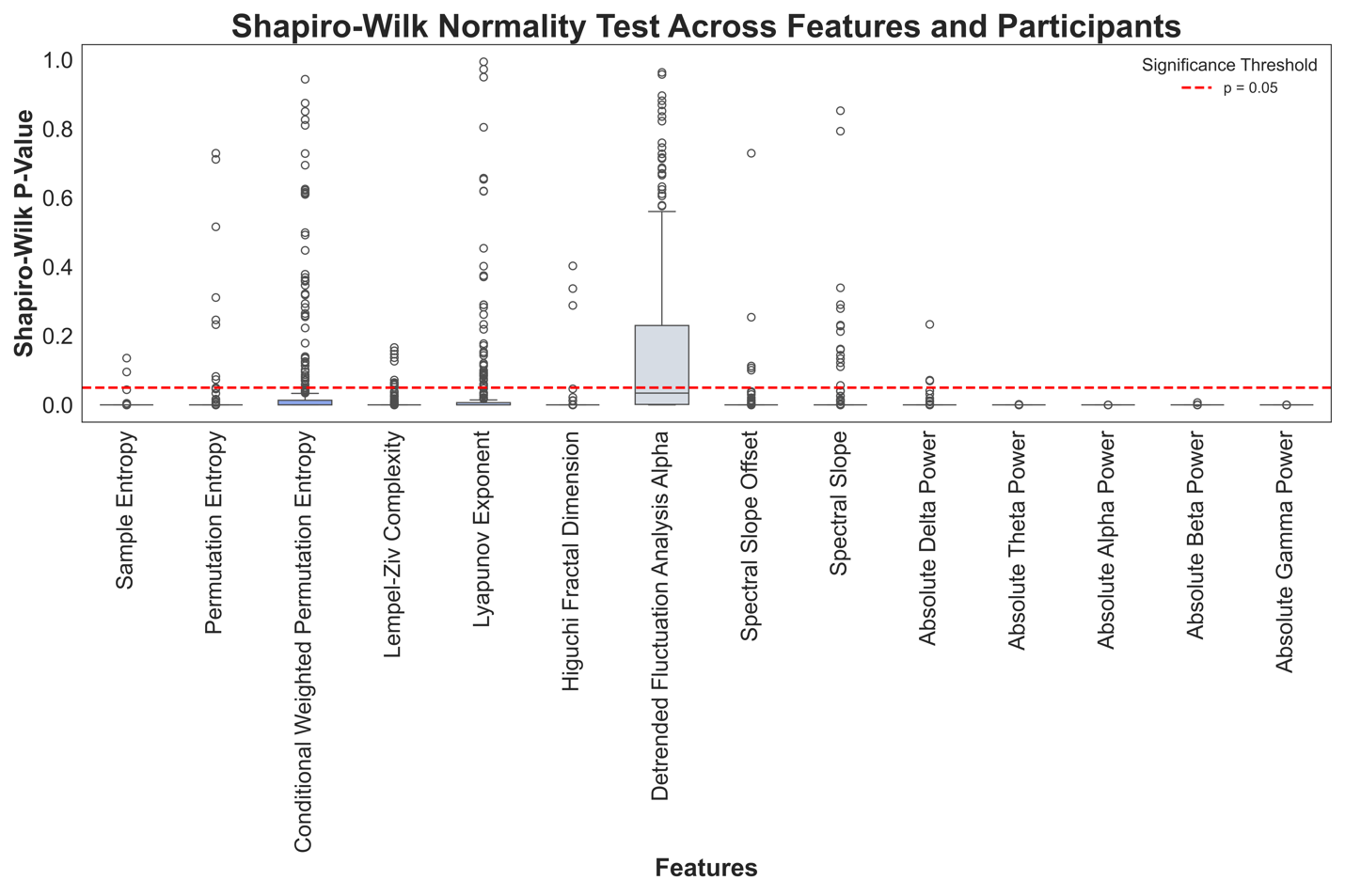


Each point represents the Shapiro-Wilk p-value for an individual EEG recording across various features, assessing the normality of the data distribution. The boxplots indicate the distribution of p-values for each feature, with the line inside each box denoting the median and the box boundaries representing the interquartile range (IQR). Whiskers extend to 1.5 times the IQR, and outliers are displayed as dots. Features with p-values less than 0.05 (below the red dashed line) suggest the data significantly deviates from normality.

**Figure S2**

**
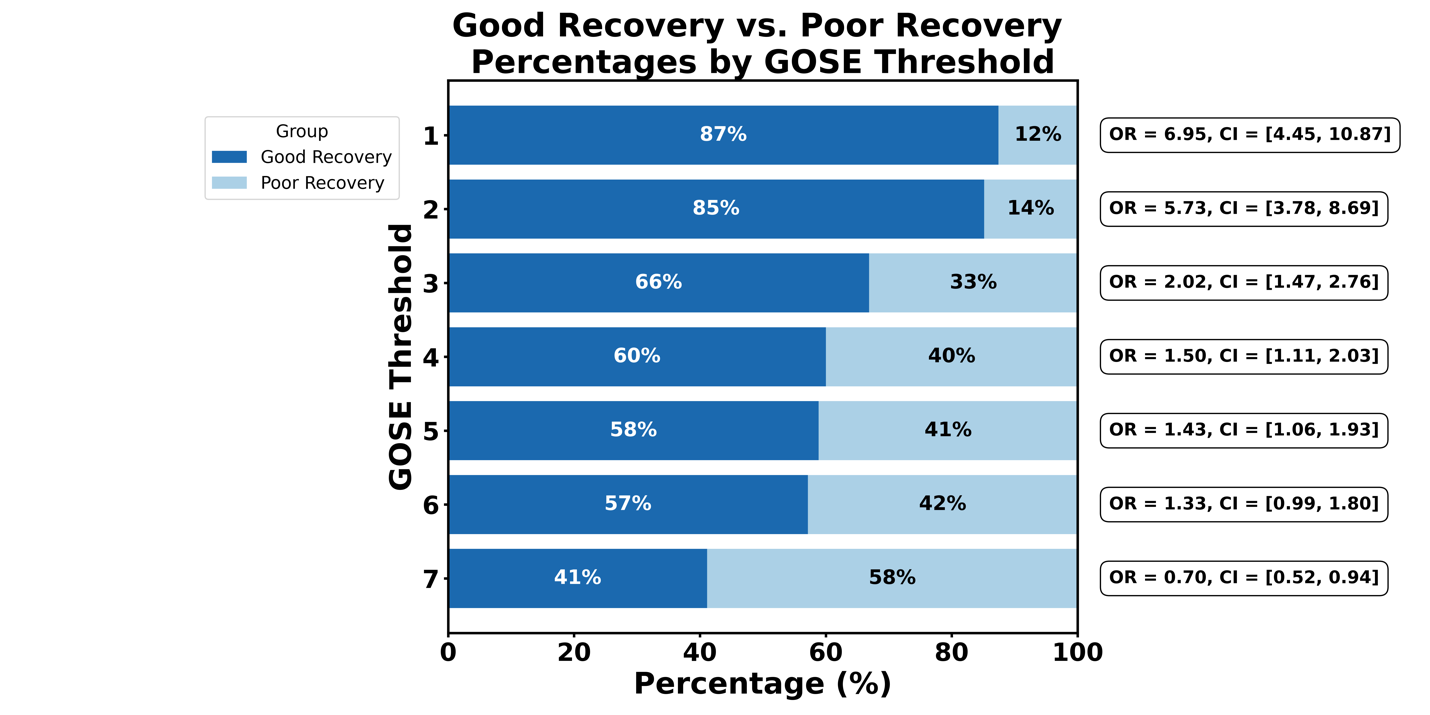
**

Ordinal Ratio Analysis: Percentage distribution of patients with good recovery and poor recovery across different GOS-E thresholds (1–7). Odds ratios (OR) with 95% confidence intervals (CI) for each threshold to evaluate group balance.

**Table S1**

Mann-Whitney U-Statistics

| GOSE Threshold | Conditional Weighted Permutation Entropy | Spectral Slope Offset | Spectral Slope | Higuchi Fractal Dimension | Lyapunov Exponent | Lempel-Ziv Complexity | Permutation Entropy | Sample Entropy | Detrended Fluctuation Analysis Alpha | Absolute Alpha Power | Absolute Beta Power | Absolute Delta Power | Absolute Gamma Power | Absolute Theta Power |
| --- | --- | --- | --- | --- | --- | --- | --- | --- | --- | --- | --- | --- | --- | --- |
| 1 | 1794.0 | 2023.0 | 2112.0 | 1275.0 | 1318.0 | 1649.5 | 1430.0 | 1099.0 | 2307.0 | 1364.0 | 1703.0 | 1554.0 | 1096.0 | 988.0 |
| 2 | 2012.0 | 2517.0 | 2445.0 | 1750.0 | 1768.0 | 2011.0 | 1763.0 | 1320.0 | 2389.0 | 1829.0 | 2074.0 | 2020.0 | 1327.0 | 1389.0 |
| 3 | 2896.0 | 3014.0 | 2071.0 | 4523.0 | 2768.0 | 5253.5 | 4093.0 | 4151.0 | 4687.0 | 5347.0 | 5645.0 | 2981.0 | 4613.0 | 4231.0 |
| 4 | 3799.0 | 3335.0 | 2617.0 | 5176.0 | 3595.0 | 5741.0 | 4980.0 | 4201.0 | 5337.0 | 5682.0 | 5807.0 | 3278.0 | 4477.0 | 4320.0 |
| 5 | 3691.0 | 3518.0 | 2666.0 | 5360.0 | 3620.0 | 5859.0 | 4914.0 | 4221.0 | 5286.0 | 5831.0 | 5937.0 | 3404.0 | 4586.0 | 4510.0 |
| 6 | 3645.0 | 3756.0 | 2936.0 | 5485.0 | 3790.0 | 5792.5 | 4945.0 | 4161.0 | 5174.0 | 5901.0 | 5938.0 | 3567.0 | 4440.0 | 4646.0 |
| 7 | 3555.0 | 4019.0 | 2932.0 | 5318.0 | 3712.0 | 5138.5 | 4760.0 | 4364.0 | 4365.0 | 5369.0 | 5393.0 | 4299.0 | 4624.0 | 5090.0 |

Mann-Whitney P-Values

| GOSE Threshold | Conditional Weighted Permutation Entropy | Spectral Slope Offset | Spectral Slope | Higuchi Fractal Dimension | Lyapunov Exponent | Lempel-Ziv Complexity | Permutation Entropy | Sample Entropy | Detrended Fluctuation Analysis Alpha | Absolute Alpha Power | Absolute Beta Power | Absolute Delta Power | Absolute Gamma Power | Absolute Theta Power |
| --- | --- | --- | --- | --- | --- | --- | --- | --- | --- | --- | --- | --- | --- | --- |
| 1 | 6.190e-01 | 1.265e-01 | 5.379e-02 | 6.665e-02 | 1.009e-01 | 8.819e-01 | 2.558e-01 | *8.636e-03 | 5.013e-03 | 1.517e-01 | 9.301e-01 | 5.630e-01 | 8.299e-03 | 1.774e-03 |
| 2 | 7.546e-01 | *1.505e-02 | 3.325e-02 | 4.340e-01 | 4.796e-01 | 7.577e-01 | 4.667e-01 | 9.700e-03 | 5.821e-02 | 6.520e-01 | 5.669e-01 | 7.293e-01 | 1.056e-02 | 2.163e-02 |
| 3 | *1.155e-01 | 2.302e-01 | *2.804e-05 | 3.433e-04 | *4.776e-02 | 3.687e-09 | 2.661e-02 | 1.635e-02 | 4.130e-05 | 5.935e-10 | *9.558e-13 | 1.921e-01 | *1.108e-04 | 7.938e-03 |
| 4 | 7.068e-01 | 3.011e-01 | 1.278e-03 | 4.875e-06 | 8.087e-01 | 3.115e-10 | *7.093e-05 | 1.095e-01 | *4.182e-07 | 9.886e-10 | 8.473e-11 | 2.272e-01 | 1.464e-02 | 4.965e-02 |
| 5 | 9.601e-01 | 5.656e-01 | 1.589e-03 | 5.512e-07 | 7.908e-01 | *6.906e-11 | 2.569e-04 | 1.202e-01 | 1.725e-06 | 1.229e-10 | 1.408e-11 | 3.574e-01 | 7.798e-03 | 1.509e-02 |
| 6 | 7.527e-01 | 9.868e-01 | 1.418e-02 | *1.698e-07 | 9.052e-01 | 7.329e-10 | 3.163e-04 | 2.158e-01 | 1.771e-05 | *8.932e-11 | 4.237e-11 | 5.821e-01 | 3.762e-02 | 6.933e-03 |
| 7 | 6.438e-01 | 3.465e-01 | 1.870e-02 | 1.060e-06 | 9.915e-01 | 1.442e-05 | 1.431e-03 | 4.686e-02 | 4.652e-02 | 4.782e-07 | 3.262e-07 | *7.338e-02 | 5.504e-03 | *2.803e-05 |

Results of the ordinal ratio analysis: feature Mann-Whitney U-statistic and p-value determined per GOS-E threshold.

**Table S2**

| **Feature** | **Shapiro Statistic (Good Recovery)** | **Shapiro P-Value (Good Recovery)** | **Shapiro Statistic (Poor Recovery)** | **Shapiro P-Value (Poor Recovery)** | **Levene Statistic** | **Levene P-Value** |
| --- | --- | --- | --- | --- | --- | --- |
| **Sample Entropy** | 0.822820 | 1.511331e-10 | 0.586992 | 1.624771e-11 | 2.181062 | 1.415360e-01 |
| **Permutation Entropy** | 0.976918 | 4.113821e-02 | 0.986879 | 7.838594e-01 | 0.298413 | 5.855826e-01 |
| **Conditional Weighted Permutation Entropy** | 0.946222 | 1.417147e-04 | 0.866531 | 1.306786e-05 | 32.148596 | 5.879422e-08 |
| **Lempel-Ziv Complexity** | 0.973850 | 2.185747e-02 | 0.698164 | 1.259616e-09 | 0.352556 | 5.534451e-01 |
| **Lyapunov Exponent** | 0.848669 | 1.384120e-09 | 0.870299 | 1.712753e-05 | 6.170553 | 1.394092e-02 |
| **Higuchi Fractal Dimension** | 0.972044 | 1.514532e-02 | 0.935910 | 4.311458e-03 | 1.580438 | 2.103922e-01 |
| **Detrended Fluctuation Analysis Alpha** | 0.953273 | 4.573467e-04 | 0.964158 | 8.434064e-02 | 0.306914 | 5.802954e-01 |
| **Spectral Slope Offset** | 0.968361 | 7.279522e-03 | 0.851634 | 4.659591e-06 | 0.182107 | 6.700995e-01 |
| **Spectral Slope** | 0.983609 | 1.656097e-01 | 0.864714 | 1.148641e-05 | 37.819721 | 5.216189e-09 |
| **Absolute Delta Power** | 0.968153 | 6.989036e-03 | 0.965384 | 9.652867e-02 | 0.003182 | 9.550775e-01 |
| **Absolute Theta Power** | 0.835956 | 4.526897e-10 | 0.675390 | 4.777084e-10 | 1.815468 | 1.796158e-01 |
| **Absolute Alpha Power** | 0.805695 | 3.909886e-11 | 0.613235 | 4.191379e-11 | 6.760297 | 1.012667e-02 |
| **Absolute Beta Power** | 0.707328 | 5.833604e-14 | 0.535950 | 2.879949e-12 | 7.549303 | 6.639556e-03 |
| **Absolute Gamma Power** | 0.857179 | 3.026420e-09 | 0.435602 | 1.369868e-13 | 7.514235 | 6.764466e-03 |

Shapiro-Wilk and Levene’s test results for EEG features across recovery groups (GOSE ≥4 threshold for recovery). For each feature, the Shapiro-Wilk statistic and corresponding p-value are displayed for both recovered and non-recovered, alongside Levene's test statistic and p-value for variance homogeneity between groups.

**Figure S3**

**
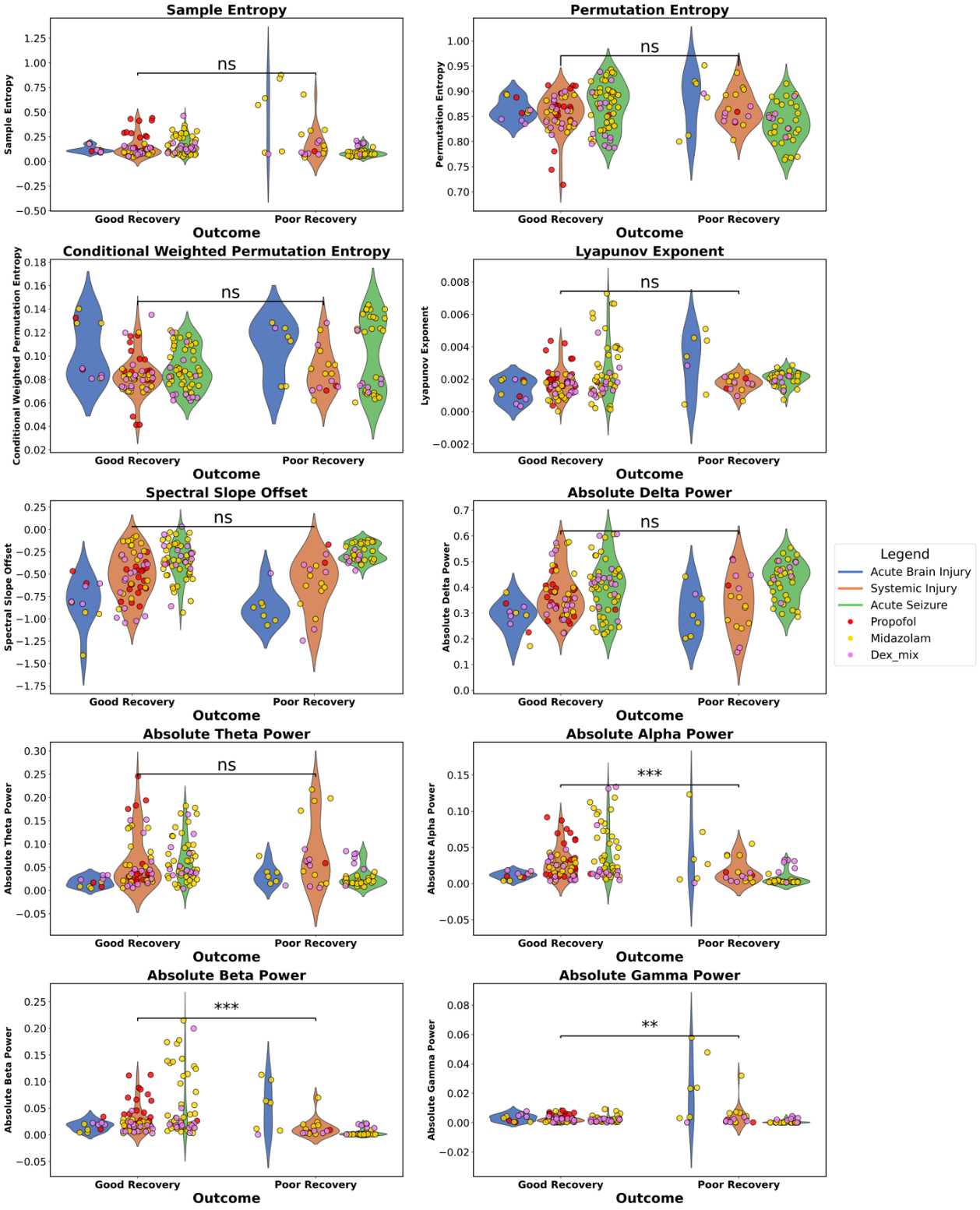
**

Feature group differences determined by Mann-Whitney U tests (with Bonferroni correction for multiple comparisons) plotted with significance (* p>=0.05, ** p>=0.01, *** p>= 0.001). For visualization, groups are split by etiologies in violin plots: blue (acute brain injury), orange (systemic injury), green (acute seizure). Within each etiological subgroup, scatter plots represent sedatives: red (propofol), yellow (midazolam), purple (dexmedetomidine with a GABAergic sedative).

**Figure S4**


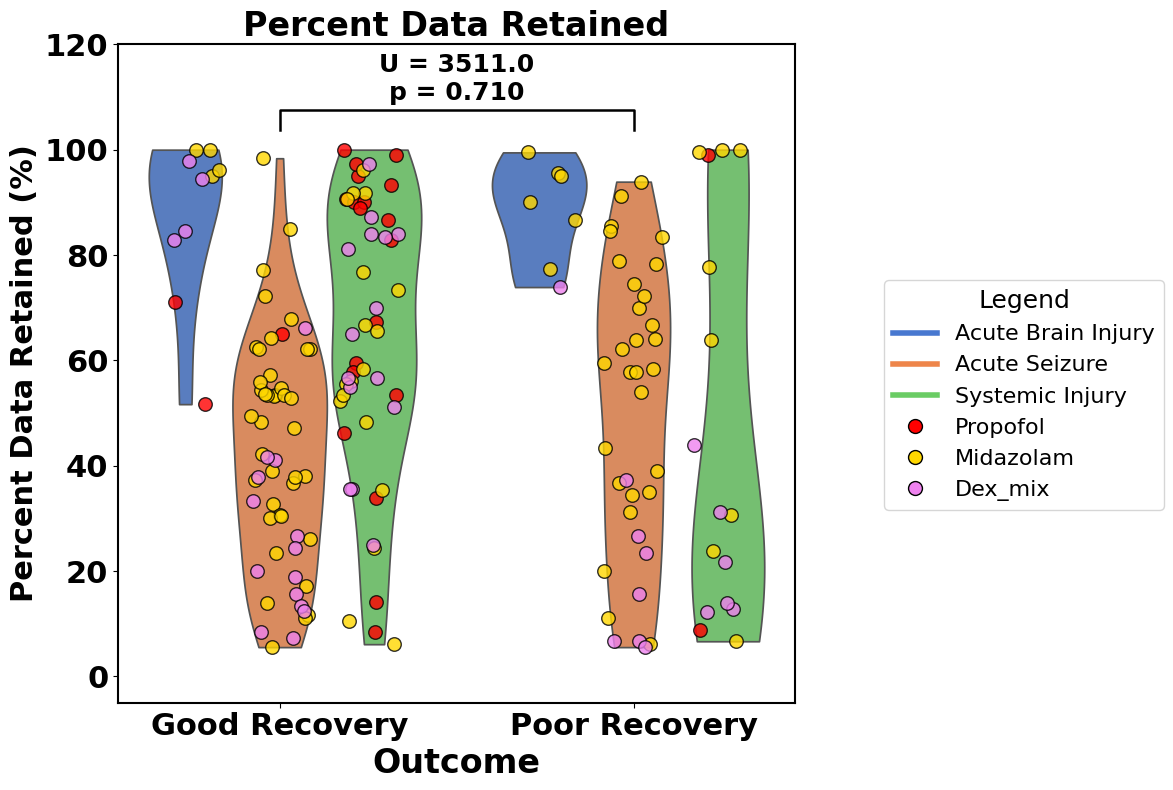


Percent EEG data retained across functional outcome groups. Violin plots show the distribution of the percentage of EEG data retained after preprocessing and quality control for patients with good versus poor functional recovery. Functional outcome was defined using 3-month Glasgow Outcome Scale–Extended scores, with Good Recovery defined as GOSE ≥ 4 and Poor Recovery defined as GOSE < 4. For visualization, groups are split by etiologies represented as violin plots: acute brain injury (blue), systemic injury (orange), acute seizure (green). Within each etiological subgroup, the scatter plots represent sedative agent: propofol (red), midazolam (yellow), dexmedetomidine with a GABAergic sedative (purple). A Mann–Whitney U test showed no significant difference in retained data between outcome groups (U = 3511.0, p = 0.710), indicating that differences in outcome-related analyses are unlikely to be explained by unequal data retention across groups.

**Table S3**

| **Condition** | **Feature** | **U Statistic** | **Median Good Recovery** | **Median Poor Recovery** | **P Value** | **Significance** |
| --- | --- | --- | --- | --- | --- | --- |
| Midazolam | Absolute Alpha Power | 2160 | 0.025 | 0.003 | <0.001 | *** |
| Midazolam | Absolute Beta Power | 2185 | 0.020 | 0.002 | <0.001 | *** |
| Midazolam | Absolute Delta Power | 1283 | 0.377 | 0.368 | 1.000 | ns |
| Midazolam | Absolute Gamma Power | 1854 | 0.001 | 0.000 | 0.011 | * |
| Midazolam | Absolute Theta Power | 1799 | 0.052 | 0.020 | 0.042 | * |
| Midazolam | Conditional Weighted Permutation Entropy | 1002 | 0.084 | 0.115 | 1.000 | ns |
| Midazolam | Detrended Fluctuation Analysis Alpha | 2119 | 0.667 | 0.589 | <0.001 | *** |
| Midazolam | Higuchi Fractal Dimension | 1880 | 1.918 | 1.894 | 0.005 | ** |
| Midazolam | Lempel-Ziv Complexity | 2074.5 | 0.228 | 0.119 | <0.001 | *** |
| Midazolam | Lyapunov Exponent | 1096 | 0.002 | 0.002 | 1.000 | ns |
| Midazolam | Permutation Entropy | 1699 | 0.877 | 0.845 | 0.362 | ns |
| Midazolam | Sample Entropy | 1562 | 0.109 | 0.088 | 1.000 | ns |
| Midazolam | Spectral Slope | 758 | 2.400 | 3.131 | 0.013 | * |
| Midazolam | Spectral Slope Offset | 1330 | -0.344 | -0.330 | 1.000 | ns |
| Propofol | Absolute Alpha Power | 30 | 0.027 | 0.014 | 1.000 | ns |
| Propofol | Absolute Beta Power | 44 | 0.039 | 0.007 | 0.304 | ns |
| Propofol | Absolute Delta Power | 3 | 0.349 | 0.460 | 1.000 | ns |
| Propofol | Absolute Gamma Power | 39 | 0.003 | 0.001 | 1.000 | ns |
| Propofol | Absolute Theta Power | 8 | 0.032 | 0.060 | 1.000 | ns |
| Propofol | Conditional Weighted Permutation Entropy | 38 | 0.087 | 0.073 | 1.000 | ns |
| Propofol | Detrended Fluctuation Analysis Alpha | 24 | 0.677 | 0.677 | 1.000 | ns |
| Propofol | Higuchi Fractal Dimension | 26 | 1.913 | 1.911 | 1.000 | ns |
| Propofol | Lempel-Ziv Complexity | 35 | 0.250 | 0.172 | 1.000 | ns |
| Propofol | Lyapunov Exponent | 29 | 0.002 | 0.002 | 1.000 | ns |
| Propofol | Permutation Entropy | 33 | 0.869 | 0.848 | 1.000 | ns |
| Propofol | Sample Entropy | 39 | 0.140 | 0.085 | 1.000 | ns |
| Propofol | Spectral Slope | 4 | 2.052 | 2.646 | 1.000 | ns |
| Propofol | Spectral Slope Offset | 2 | -0.553 | -0.271 | 1.000 | ns |
| Dex_mix | Absolute Alpha Power | 278 | 0.014 | 0.013 | 1.000 | ns |
| Dex_mix | Absolute Beta Power | 335 | 0.018 | 0.007 | 0.674 | ns |
| Dex_mix | Absolute Delta Power | 166 | 0.354 | 0.444 | 1.000 | ns |
| Dex_mix | Absolute Gamma Power | 271 | 0.002 | 0.002 | 1.000 | ns |
| Dex_mix | Absolute Theta Power | 194 | 0.038 | 0.061 | 1.000 | ns |
| Dex_mix | Conditional Weighted Permutation Entropy | 222 | 0.081 | 0.076 | 1.000 | ns |
| Dex_mix | Detrended Fluctuation Analysis Alpha | 201 | 0.626 | 0.639 | 1.000 | ns |
| Dex_mix | Higuchi Fractal Dimension | 225 | 1.897 | 1.907 | 1.000 | ns |
| Dex_mix | Lempel-Ziv Complexity | 312 | 0.195 | 0.166 | 1.000 | ns |
| Dex_mix | Lyapunov Exponent | 137 | 0.001 | 0.002 | 1.000 | ns |
| Dex_mix | Permutation Entropy | 214 | 0.842 | 0.850 | 1.000 | ns |
| Dex_mix | Sample Entropy | 199 | 0.124 | 0.159 | 1.000 | ns |
| Dex_mix | Spectral Slope | 177 | 2.298 | 2.392 | 1.000 | ns |
| Dex_mix | Spectral Slope Offset | 222 | -0.398 | -0.385 | 1.000 | ns |

Group comparisons of EEG features across conditions and recovery status. Mann-Whitney U tests (with Bonferroni correction for multiple comparisons) for group comparisons of EEG features between good recovery and poor recovery under three sedative conditions (propofol, midazolam and dex_mix). For midazolam, there were 62 files from 12 recovered patients and 42 files from 8 non-recovered patients. For propofol, there were 22 files from 8 good recovery patients and 2 files from 2 poor recovery. For dexmedetomidine with a GABAergic sedative, there were 33 files from 11 good recovery patients and 14 files from 5 poor recovery patients. (*p < 0.05, **p < 0.01, ***p < 0.001).

**Table S4**

| **Etiology** | **Feature** | **U Statistic** | **Median Good Recovery** | **Median Poor Recovery** | **P Value** | **Significance** |
| --- | --- | --- | --- | --- | --- | --- |
| Acute Brain Injury | Absolute Alpha Power | 26 | 0.011 | 0.027 | 1 | ns |
| Acute Brain Injury | Absolute Beta Power | 24 | 0.019 | 0.060 | 1 | ns |
| Acute Brain Injury | Absolute Delta Power | 32 | 0.297 | 0.292 | 1 | ns |
| Acute Brain Injury | Absolute Gamma Power | 21 | 0.004 | 0.023 | 1 | ns |
| Acute Brain Injury | Absolute Theta Power | 18 | 0.018 | 0.030 | 1 | ns |
| Acute Brain Injury | Conditional Weighted Permutation Entropy | 38 | 0.089 | 0.116 | 1 | ns |
| Acute Brain Injury | Detrended Fluctuation Analysis Alpha | 60 | 0.705 | 0.633 | 0.570 | ns |
| Acute Brain Injury | Higuchi Fractal Dimension | 18 | 1.892 | 1.936 | 1 | ns |
| Acute Brain Injury | Lempel-Ziv Complexity | 24 | 0.241 | 0.442 | 1 | ns |
| Acute Brain Injury | Lyapunov Exponent | 15 | 0.001 | 0.003 | 1 | ns |
| Acute Brain Injury | Permutation Entropy | 22 | 0.859 | 0.895 | 1 | ns |
| Acute Brain Injury | Sample Entropy | 27 | 0.111 | 0.572 | 1 | ns |
| Acute Brain Injury | Spectral Slope | 52 | 1.934 | 1.325 | 1 | ns |
| Acute Brain Injury | Spectral Slope Offset | 48 | -0.813 | -0.872 | 1 | ns |
| Acute Seizure | Absolute Alpha Power | 1736 | 0.022 | 0.003 | <0.001 | *** |
| Acute Seizure | Absolute Beta Power | 1808 | 0.024 | 0.001 | <0.001 | *** |
| Acute Seizure | Absolute Delta Power | 753 | 0.409 | 0.439 | 1 | ns |
| Acute Seizure | Absolute Gamma Power | 1657 | 0.002 | 0.000 | <0.001 | *** |
| Acute Seizure | Absolute Theta Power | 1510 | 0.048 | 0.019 | <0.001 | *** |
| Acute Seizure | Conditional Weighted Permutation Entropy | 771 | 0.087 | 0.121 | 1 | ns |
| Acute Seizure | Detrended Fluctuation Analysis Alpha | 1299 | 0.641 | 0.572 | 0.228 | ns |
| Acute Seizure | Higuchi Fractal Dimension | 1531 | 1.932 | 1.894 | <0.001 | *** |
| Acute Seizure | Lempel-Ziv Complexity | 1793 | 0.239 | 0.119 | <0.001 | *** |
| Acute Seizure | Lyapunov Exponent | 900 | 0.002 | 0.002 | 1 | ns |
| Acute Seizure | Permutation Entropy | 1364 | 0.877 | 0.841 | 0.038 | * |
| Acute Seizure | Sample Entropy | 1452 | 0.134 | 0.079 | 0.002 | ** |
| Acute Seizure | Spectral Slope | 250 | 2.375 | 3.148 | <0.001 | *** |
| Acute Seizure | Spectral Slope Offset | 694 | -0.340 | -0.286 | 1 | ns |
| Systemic Injury | Absolute Alpha Power | 567 | 0.025 | 0.011 | 1 | ns |
| Systemic Injury | Absolute Beta Power | 632 | 0.020 | 0.007 | 0.077 | ns |
| Systemic Injury | Absolute Delta Power | 475 | 0.354 | 0.345 | 1 | ns |
| Systemic Injury | Absolute Gamma Power | 401 | 0.002 | 0.002 | 1 | ns |
| Systemic Injury | Absolute Theta Power | 342 | 0.036 | 0.060 | 1 | ns |
| Systemic Injury | Conditional Weighted Permutation Entropy | 376 | 0.082 | 0.085 | 1 | ns |
| Systemic Injury | Detrended Fluctuation Analysis Alpha | 527 | 0.673 | 0.623 | 1 | ns |
| Systemic Injury | Higuchi Fractal Dimension | 479 | 1.907 | 1.905 | 1 | ns |
| Systemic Injury | Lempel-Ziv Complexity | 526.5 | 0.217 | 0.190 | 1 | ns |
| Systemic Injury | Lyapunov Exponent | 393 | 0.002 | 0.002 | 1 | ns |
| Systemic Injury | Permutation Entropy | 395 | 0.862 | 0.858 | 1 | ns |
| Systemic Injury | Sample Entropy | 389 | 0.105 | 0.116 | 1 | ns |
| Systemic Injury | Spectral Slope | 421 | 2.186 | 2.337 | 1 | ns |
| Systemic Injury | Spectral Slope Offset | 487 | -0.481 | -0.560 | 1 | ns |

Group comparisons of EEG features across conditions and recovery status. Mann-Whitney U tests (with Bonferroni correction for multiple comparisons) for group comparisons of EEG features between good recovery and poor recovery under three etiologies (acute brain injury, systemin injury and acute seizure). For acute brain injury, there were 10 files from 5 good recovery patients and 7 files from 2 poor recovery patients. For systemic injury, there were 52 files from 7 good recovery patients and 16 files from 6 poor recovery. For acute seizure, there were 55 files from 7 good recovery patients and 35 files from 2 poor recovery patients. (*p < 0.05, **p < 0.01, ***p < 0.001).

**Table S5**

| Full Models | | | | | |
| --- | --- | --- | --- | --- | --- |
| Logistic Regression: EEG Features, Etiology, Sex and Age | | | | | |
|  |  | Precision | Recall | F1-Score | Support |
|  | Poor Recovery | 0.74 | 0.67 | 0.70 | 58 |
|  | Good Recovery | 0.84 | 0.88 | 0.86 | 117 |
|  | Accuracy |  |  | 0.81 | 175 |
|  | Macro Average | 0.79 | 0.78 | 0.78 | 175 |
|  | Weighted Average | 0.81 | 0.81 | 0.81 | 175 |
| Linear Discriminant Analysis: EEG Features, Etiology, Sex and Age | | | | | |
|  |  | Precision | Recall | F1-Score | Support |
|  | Poor Recovery | 0.79 | 0.64 | 0.70 | 58 |
|  | Good Recovery | 0.84 | 0.91 | 0.87 | 117 |
|  | Accuracy |  |  | 0.82 | 175 |
|  | Macro Average | 0.81 | 0.78 | 0.79 | 175 |
|  | Weighted Average | 0.82 | 0.82 | 0.82 | 175 |
| Support Vector Machine: EEG Features, Etiology, Sex and Age | | | | | |
|  |  | Precision | Recall | F1-Score | Support |
|  | Poor Recovery | 0.97 | 0.62 | 0.76 | 58 |
|  | Good Recovery | 0.84 | 0.99 | 0.91 | 117 |
|  | Accuracy |  |  | 0.81 | 175 |
|  | Macro Average | 0.91 | 0.81 | 0.83 | 175 |
|  | Weighted Average | 0.88 | 0.87 | 0.86 | 175 |
| Control Models | | | | | |
| Logistic Regression: Etiology, Sex and Age | | | | | |
|  |  | Precision | Recall | F1-Score | Support |
|  | Poor Recovery | 0.72 | 0.50 | 0.59 | 58 |
|  | Good Recovery | 0.79 | 0.91 | 0.84 | 117 |
|  | Accuracy |  |  | 0.81 | 175 |
|  | Macro Average | 0.76 | 0.70 | 0.72 | 175 |
|  | Weighted Average | 0.77 | 0.77 | 0.76 | 175 |
| Logistic Regression: Etiology | | | | | |
|  |  | Precision | Recall | F1-Score | Support |
|  | Poor Recovery | 0 | 0 | 0 | 58 |
|  | Good Recovery | 0.67 | 1 | 0.80 | 117 |
|  | Accuracy |  |  | 0.67 | 175 |
|  | Macro Average | 0.33 | 0.5 | 0.4 | 175 |
|  | Weighted Average | 0.45 | 0.67 | 0.54 | 175 |
| Logistic Regression: Sex | | | | | |
|  |  | Precision | Recall | F1-Score | Support |
|  | Poor Recovery | 0 | 0 | 0 | 58 |
|  | Good Recovery | 0.67 | 1 | 0.80 | 117 |
|  | Accuracy |  |  | 0.67 | 175 |
|  | Macro Average | 0.33 | 0.5 | 0.4 | 175 |
|  | Weighted Average | 0.45 | 0.67 | 0.54 | 175 |
| Logistic Regression: Age | | | | | |
|  |  | Precision | Recall | F1-Score | Support |
|  | Poor Recovery | 0.33 | 0.02 | 0.03 | 58 |
|  | Good Recovery | 0.67 | 0.98 | 0.80 | 117 |
|  | Accuracy |  |  | 0.66 | 175 |
|  | Macro Average | 0.5 | 0.5 | 0.41 | 175 |
|  | Weighted Average | 0.56 | 0.66 | 0.54 | 175 |
| BootStrap Model (1,000 Iterations) | | | | | |
|  |  | Precision | Recall | F1-Score | Support |
|  | Poor Recovery | 0.815 (±0.047) | 0.807 (±0.052) | 0.81 (±0.043) | 58 |
|  | Good Recovery | 0.81 (±0.046) | 0.815 (±0.053) | 0.811 (±0.053) | 58 |
|  | Accuracy |  |  | 0.811 (±0.043) | 116 |
|  | Macro Average | 0.812 (±0.043) | 0.811 (±0.043) | 0.811 (±0.043) | 116 |
|  | Weighted Average | 0.812 (±0.043) | 0.811 (±0.043) | 0.811 (±0.043) | 116 |

Classification reports for machine learning models including logistic regression, LDA, and SVM models across different feature sets. Full models incorporate EEG features with etiology, sex, and age as input features. The results are summarized for each classifier (Logistic Regression, LDA, and SVM). Control models evaluate the influence of non-EEG features by progressively limiting the input features to etiology, sex, and age using a Logistic Regression classifier and a bootstrap analysis that was conducted over 1,000 iterations to assess the variability and stability of model performance using resampled datasets.

**Table S6**

| **Participant** | **Age** | **Sex** | **Etiology** | **Dose Summary** |
| --- | --- | --- | --- | --- |
| sub-003AC | 8 | Male | Acute Brain Injury | 1) Hydro. 10 + Prop. 6; 2) Hydro. 8 + Prop. 6 |
| sub-006MC | 9 | Male | Systemic Injury | 1) Fent. 2 + Prop. 2; 2) Fent. 2 + Prop. 3; 3) Fent. 2 + Prop. 4; 4) Prop. 2; 5) Prop. 4 |
| sub-008MC | 16 | Male | Systemic Injury | 1) Fent. 4 + Dex. 0.2 |
| sub-009AC | 11 | Male | Systemic Injury | 1) Dex. 0.5; 2) Hydro. 4 + Dex. 0.5; 3) Hydro. 4 + Dex. 0.5 + Prop. 3; 4) Prop. 3 |
| sub-011AC | 11 | Female | Systemic Injury | 1) Dex. 0.7 + Mid. 5; 2) Hydro. 2 + Dex. 0.7 + Mid. 5; 3) Hydro. 2 + Mid. 5 |
| sub-013AC | 7 | Female | Systemic Injury | 1) Mid. 2 |
| sub-013MC | 8 | Female | Acute Seizure | 1) Mid. 1; 2) Mid. 3; 3) Mid. 4; 4) Mid. 6 |
| sub-014MC | 11 | Male | Systemic Injury | 1) Mid. 6 |
| sub-015MC | 12 | Male | Acute Seizure | 1) Mid. 1; 2) Mid. 2; 3) Mid. 4; 4) Mid. 5 |
| sub-016MC | 13 | Male | Acute Seizure | 1) Dex. 0.3; 2) Mid. 10; 3) Mid. 11; 4) Mid. 12; 5) Mid. 14; 6) Mid. 16; 7) Mid. 18; 8) Mid. 1; 9) Mid. 20; 10) Mid. 22; 11) Mid. 24; 12) Mid. 2; 13) Mid. 3; 14) Mid. 4; 15) Mid. 5; 16) Mid. 6; 17) Mid. 7; 18) Mid. 8; 19) Mid. 9 |
| sub-017MC | 11 | Female | Acute Brain Injury | 1) Mid. 2 |
| sub-018MC | 6 | Male | Acute Seizure | 1) Mid. 2 |
| sub-019MC | 6 | Female | Acute Seizure | 1) Mid. 10; 2) Mid. 12; 3) Mid. 16; 4) Mid. 18; 5) Mid. 2; 6) Mid. 4; 7) Mid. 6; 8) Mid. 8 |
| sub-020MC | 13 | Male | Acute Seizure | 1) Mid. 1; 2) Mid. 2; 3) Mid. 3; 4) Mid. 4; 5) Mid. 5; 6) Mid. 6 |
| sub-021MC | 13 | Female | Systemic Injury | 1) Mid. 1; 2) Mid. 2; 3) Mid. 3 |
| sub-022MC | 12 | Female | Systemic Injury | 1) Prop. 1 |
| sub-025MC | 14 | Male | Acute Brain Injury | 1) Fent. 2 + Mid. 1 |
| sub-026MC | 16 | Male | Systemic Injury | 1) Mid. 1; 2) Mid. 2 |
| sub-027MC | 14 | Female | Systemic Injury | 1) Mid. 2; 2) Fent. 2 + Mid. 2; 3) Fent. 2 + Mid. 3 |
| sub-028MC | 6 | Male | Systemic Injury | 1) Mid. 1; 2) Prop. 2; 3) Prop. 3; 4) Dex. 0.25 + Prop. 4 |
| sub-029MC | 16 | Female | Acute Brain Injury | 1) Mid. 2; 2) Mid. 3 |
| sub-030MC | 15 | Female | Acute Brain Injury | 1) Dex. 0.5 + Prop. 1; 2) Mid. 2; 3) Mid. 4 |
| sub-031MC | 9 | Male | Acute Brain Injury | 1) Dex. 0.5; 2) Fent. 4 + Dex. 0.5; 3) Dex. 1.4; 4) Dex. 1 |
| sub-032MC | 14 | Female | Systemic Injury | 1) Dex. 0.5; 2) Dex. 0.5 + Mid. 2; 3) Dex. 0.75; 4) Prop. 2; 5) Prop. 3 |
| sub-033MC | 8 | Male | Acute Seizure | 1) Prop. 1 |
| sub-034MC | 8 | Female | Acute Seizure | 1) Mid. 10; 2) Mid. 12; 3) Mid. 14; 4) Mid. 2; 5) Mid. 3; 6) Mid. 4; 7) Mid. 6; 8) Mid. 8 |
| sub-035MC | 16 | Male | Systemic Injury | 1) Mid. 10; 2) Mid. 10 + Prop. 2; 3) Mid. 3; 4) Mid. 5; 5) Mid. 5 + Prop. 2; 6) Fent. 2 + Prop. 0.5; 7) Fent. 2 + Prop. 1 |
| sub-037MC | 14 | Female | Systemic Injury | 1) Mid. 10; 2) Mid. 12; 3) Mid. 15; 4) Mid. 1; 5) Mid. 2; 6) Mid. 3; 7) Mid. 4; 8) Mid. 6; 9) Mid. 8; 10) Prop. 3; 11) Prop. 4.8; 12) Prop. 4 |
| sub-038MC | 15 | Male | Acute Brain Injury | 1) Fent. 4 + Mid. 2 |
| sub-039MC | 11 | Male | Systemic Injury | 1) Fent. 2 + Dex. 0.5 |
| sub-040MC | 10 | Female | Systemic Injury | 1) Prop. 4 |
| sub-041MC | 10 | Male | Acute Seizure | 1) Mid. 0.5; 2) Mid. 1.5; 3) Mid. 10; 4) Mid. 12; 5) Mid. 16; 6) Mid. 18; 7) Mid. 2.5; 8) Mid. 20; 9) Mid. 22; 10) Mid. 24; 11) Mid. 24 + Prop. 1; 12) Fent. 1 + Mid. 24 + Prop. 1; 13) Fent. 3 + Mid. 25 + Prop. 2; 14) Mid. 2; 15) Mid. 3; 16) Mid. 4; 17) Mid. 5; 18) Mid. 6 |

Participant-level medication-dose summary for analyzed sedated EEG files. Each row represents one participant. Age is reported in years. Etiology indicates the primary injury category. The dose summary lists the medication-dose combinations present during the analyzed EEG files for each participant. Numbered entries correspond to distinct analyzed file-level medication conditions. Medications joined by “+” were co-administered within the same EEG file, while semicolons separate different analyzed file-level medication-dose combinations. Drug abbreviations and units are as follows: Prop. = propofol, reported in mg/kg/hr; Mid. = midazolam, reported in mcg/kg/min; Dex. = dexmedetomidine, reported in mcg/kg/hr; Fent. = fentanyl, reported in mcg/kg/hr; and Hydro. = hydromorphone, reported in mcg/kg/hr.
